# Detecting SARS-CoV-2 variants with SNP genotyping

**DOI:** 10.1371/journal.pone.0243185

**Published:** 2021-02-24

**Authors:** Helen Harper, Amanda Burridge, Mark Winfield, Adam Finn, Andrew Davidson, David Matthews, Stephanie Hutchings, Barry Vipond, Nisha Jain, The COVID-19 Genomics UK (COG-UK) Consortium, Keith Edwards, Gary Barker

**Affiliations:** 1 School of Biological Sciences, University of Bristol, Bristol, United Kingdom; 2 School of Cellular and Molecular Medicine, University of Bristol, Bristol, United Kingdom; 3 PHE South West Regional Laboratory, Southmead Hospital, Bristol, United Kingdom; 4 3CR Bioscience Limited, West Point Business Park, Essex, United Kingdom; "INSERM", FRANCE

## Abstract

Tracking genetic variations from positive SARS-CoV-2 samples yields crucial information about the number of variants circulating in an outbreak and the possible lines of transmission but sequencing every positive SARS-CoV-2 sample would be prohibitively costly for population-scale test and trace operations. Genotyping is a rapid, high-throughput and low-cost alternative for screening positive SARS-CoV-2 samples in many settings. We have designed a SNP identification pipeline to identify genetic variation using sequenced SARS-CoV-2 samples. Our pipeline identifies a minimal marker panel that can define distinct genotypes. To evaluate the system, we developed a genotyping panel to detect variants-identified from SARS-CoV-2 sequences surveyed between March and May 2020 and tested this on 50 stored qRT-PCR positive SARS-CoV-2 clinical samples that had been collected across the South West of the UK in April 2020. The 50 samples split into 15 distinct genotypes and there was a 61.9% probability that any two randomly chosen samples from our set of 50 would have a distinct genotype. In a high throughput laboratory, qRT-PCR positive samples pooled into 384-well plates could be screened with a marker panel at a cost of < £1.50 per sample. Our results demonstrate the usefulness of a SNP genotyping panel to provide a rapid, cost-effective, and reliable way to monitor SARS-CoV-2 variants circulating in an outbreak. Our analysis pipeline is publicly available and will allow for marker panels to be updated periodically as viral genotypes arise or disappear from circulation.

## Introduction

In March 2020 the World Health Organisation characterised the global outbreak of COVID-19, caused by the severe acute respiratory syndrome coronavirus 2 (SARS-CoV-2), as a pandemic [1]. A huge global effort followed to learn more about the virus, how it is transmitted and the disease it causes, in order to prevent and control outbreaks and find effective treatments and vaccines.

Since the first SARS-CoV-2 genome sequence was released in January 2020, tens of thousands of genome sequences have been shared online in public databases [2, 3]. Access to sequence data is crucial for researchers to identify novel mutations, design diagnostic tests and vaccines, and to track outbreaks; allowing researchers to follow the transmission of SARS-CoV-2 both locally and globally.

As with all viruses, SARS-CoV-2 accumulates random mutations during replication. The viral replication complex has proof reading activity which may at least partially explain the relatively low rate of accumulated mutations [4]. SARS-CoV-2 accumulates mutations at about half the rate reported for influenza virus that does not have a proof-reading mechanism and likely has different structural constraints on its own proteins [5–7].

Following the emergence of SARS-CoV-2, distinct lineages have formed as viruses circulating in particular regions evolved and increased in frequency. Consortia were galvanised to sequence a large number of positive SARS-CoV-2 samples to track both the evolution and geographic movements of the virus [2, 3, 8, 9] and a nomenclature for SARS-CoV-2 lineages was suggested to enable clear communication between research groups [10].

**Fig 1:**
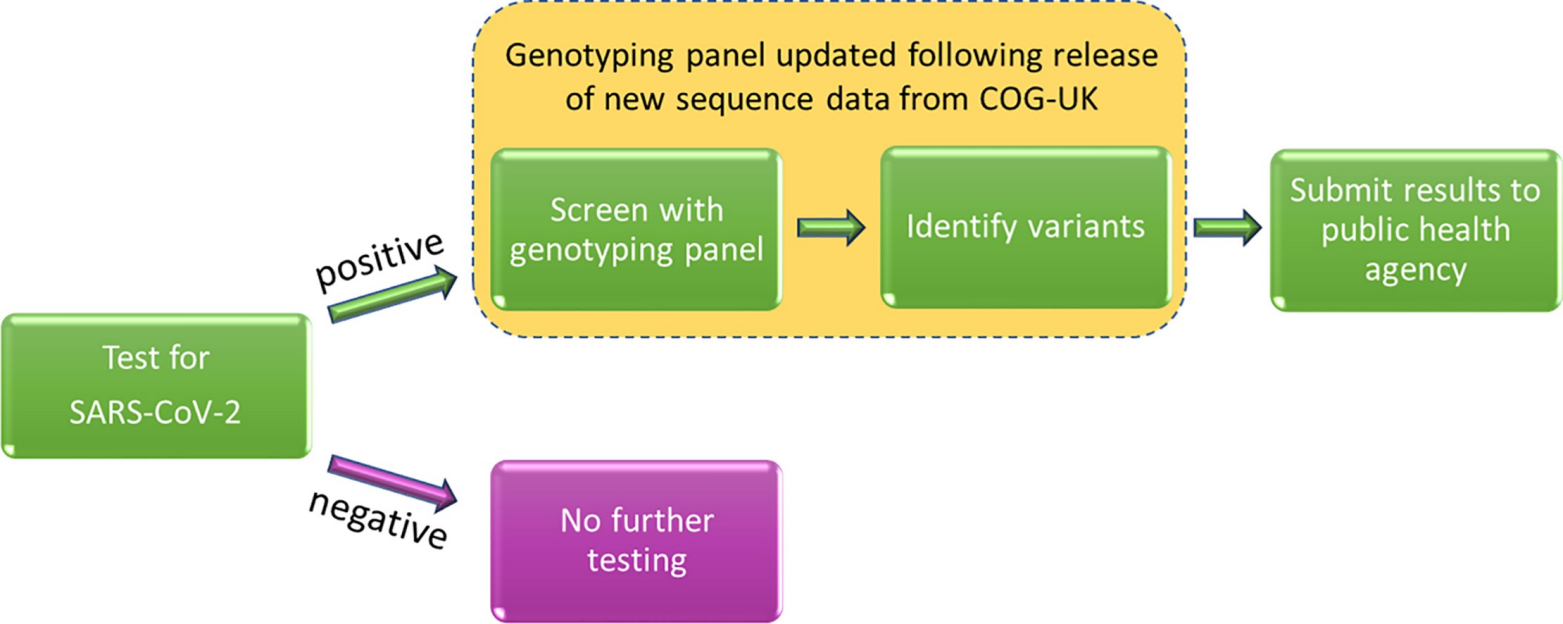
How the SARS-CoV-2 genotyping panel can be used to identify circulating SARS-CoV-2 variants.

Contact tracing procedures that utilise genomic tools have been shown to reduce the size and duration of an outbreak [11]; these tools also yield detailed information about lines of transmission. To date, SARS-CoV-2 lineages have been determined by sequencing positive SARS-CoV-2 samples. While thorough, this approach is costly and only a small proportion of positive samples have been assigned to a lineage. Our research aims to address this issue by developing a high-throughput, low-cost genotyping panel to identify circulating SARS-CoV-2 variants as genotypes. Fig 1 shows how this technique could be incorporated into diagnostic pathways. RNA from positive samples, from individuals with COVID-19, could be further screened with a SARS-CoV-2 genotyping panel to identify variants, thereby providing valuable genomic data to investigate outbreaks, potentially identifying transmission pathways linking local and regional cases and helping to inform possible interventions. Notably, efficient methods for tracking transmission of certain lineages could be vital in situations where mutations are associated with increased transmission, severity of disease or vaccine failure. We use the term genotype here as opposed to lineage as our system is designed to separate samples from an outbreak into distinct groups rather than attempt to infer their phylogenetic relationships with other samples.

We have validated this approach by genotyping positive clinical SARS-CoV-2 samples and show that this is an efficient method for assessing circulating variants in an outbreak.

## Materials and methods

### Samples

Extracted RNA from the supernatants of cultured cells infected with the laboratory cultured SARS-CoV-2 isolates GBR/Liverpool_strain/2020 and hCoV-19/England/02/2020 were used to validate the test genotyping panel (Table 1). The hCoV-19/England/02/2020 stock contained a mixture of the wild type (wt) virus and a variant with a 24 nt deletion in the spike gene as previously described [12].

**Table 1:** Laboratory samples used to validate the SARS-CoV-2 test genotyping panel.

Sample name	Source	Type	Sequenced	Spike Phenotype	Comparison to Wuhan-Hu-1 GenBank Acc: NC_045512.2 SNPs (amino acid substitutions)
GBR/liverpool_strain/2020	University of Bristol	Viral RNA isolated from cell culture supernatant.	Yes (GenBankAcc: MW041156.1)	wt spike sequence	A6948C, G11083T, C21005T, C25452T, C28253T (nsp3: N1410T, nsp6: L37F, nsp16: A116V)
hCoV-19/England/02/2020	University of Bristol	Viral RNA isolated from cell culture supernatant.	Yes (GISAID ID: EPI_ISL_407073)	Mixture* wt spike and BrisΔS	C8782T, T18488C, T23605G, T28144C, A29596G (nsp14: I150T, ORF 8: L84S, ORF 10: I13M)

*Sample known to contain wild type and deleted spike sequences (BrisΔS).

#### Clinical samples

RNA from 50 qRT-PCR positive SARS-CoV-2 samples (supplied by Public Health England, PHE) as RNA extracted from nasopharyngeal swabs were screened using the test genotyping panel.

### RNA extraction

Viral RNA was extracted from cell culture supernatants using a QIAamp Viral RNA Mini Kit (Qiagen) according to the manufacturer’s instructions.

PHE samples: Viral RNA was extracted using the silica guanidinium isothiocyanate binding method [13] adapted for the ThermoFisher Kingfisher using paramagnetic silica particles (Magnesil, Promega).

### Genotyping panel design

The trimmed SARS-CoV-2 genome sequences and related metadata were downloaded from the COVID-19 Genomics UK (COG-UK) consortium website (https://www.cogconsortium.uk/data/). To check for changes in marker frequencies between May and September 2020, both the 2020-05-08 dataset (14,277 sequences) and the updated 2020-09-03 dataset (40,640 sequences) were downloaded.

#### Marker selection

For SNP design, COG-UK consortium alignment data were pre-processed to select positions in the viral genome which were polymorphic with a minor allele frequency of > 0.001. After this step, sequenced accessions with identical genotypes across the polymorphic loci were removed to further simplify downstream analysis. Where two samples differed only at ambiguous base positions (no base pair called and thus recorded as ‘N’), they were considered as identical and only one was retained. Markers were then prioritised as follows. The SNP with the highest minor allele frequency was chosen as the first marker (the logic being that this allele will split the samples best into two groups). In subsequent steps, all remaining markers were evaluated to determine which one discriminated the maximum number of remaining unresolved sample pairs. The highest scoring SNP became marker 2 and the process iterated until either i) all samples could be separated into distinct genotypes, ii) no SNPs remained or iii) adding further SNPs did not result in the resolution of any additional sample pairs. For the final set of maximally informative SNPs, flanking sequences of 50 bases up and down-stream of the marker were extracted from the full sequence alignment (S1 Table). If polymorphisms were observed at a frequency greater than 0.5% in the flanking sequences, they were recorded as IUPAC ambiguity codes, such that they could be avoided when designing primers for the genotyping assay. The pipeline also utilised the corresponding COG-UK metadata file to assign lineages and locations to the genotypes in our analysis output files. The complete pipeline of PERL scripts along with links to example input data files is available from https://github.com/pr0kary0te/SARSmarkers.

#### Primer design

SNP coordinates and 50 bases of flanking sequence both up and downstream of it (S1 Table) were provided to 3CR Bioscience Ltd to design oligos compatible with One Step PACE-RT™ chemistry [14]. For each of the markers in the test panel, two allele-specific forward primers and one common reverse primer were designed with a PACE-specific tail (sequences available in S2 Table).

### Genotyping

**Fig 2:**
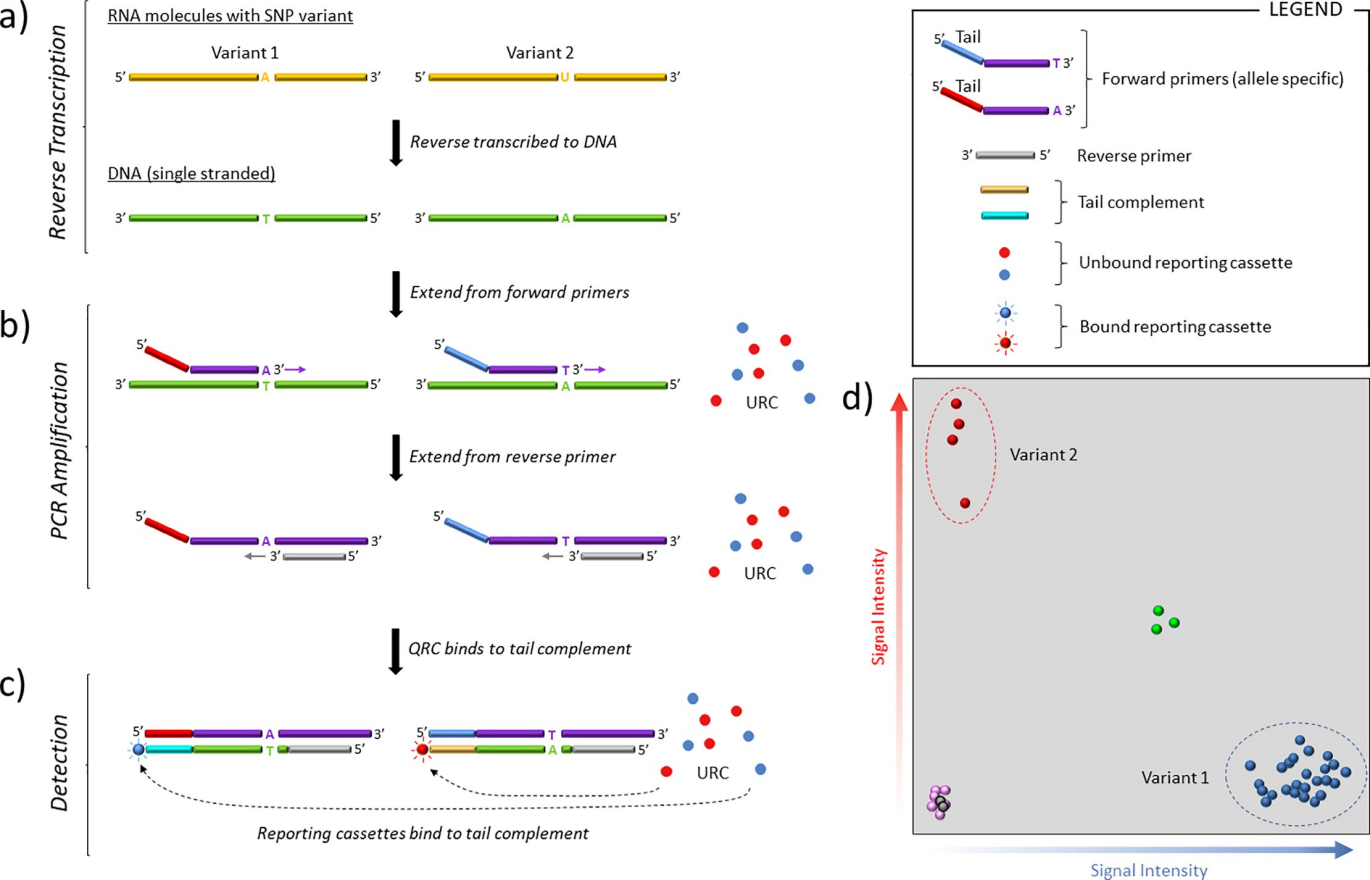
PACE-RT™ genotyping. a) The RNA sample, which here contains two SNP variants, is reverse transcribed to produce single stranded DNA. b) The target region is then amplified using two, allele specific primers which differ in their 3’ terminal base and 5’ tail, and a common reverse primer. As PCR proceeds, the tail sequences of allele-specific forward primers become incorporated in the amplified fragments and their sequence complements are generated. c) Reporting cassettes, initially quenched, bind to the appropriate tail sequence complement, become unquenched and produce a light signal (HEX (red), FAM (blue)). d) Fluorescence intensity is measured and plotted to determine allele calls. A mixed signal (green) is seen as equal signal from both fluorochromes. Samples at the origin are the no-template controls (black) and unamplified samples (pink).

Genotyping was performed using the One Step PACE-RT™ (PCR Allele Competitive Extension) kit (3CR Bioscience) scaled for 1,536 plate format, the approach is described in Fig 2.

Each One Step PACE-RT™ SNP genotyping reaction was performed using 2.5 ng RNA, 0.005 μL One Step RT-enzyme, 0.5 μL One Step PACE-RT genotyping master mix (3CR Bioscience) and 0.018 μL assay mix (12 μM of each forward primer, 30 μM reverse primer) in a total volume of 1 μL. The combined reverse transcription and DNA amplification reaction was performed using a Hydrocycler-16 (LGC Genomics, UK) under the following conditions: 50°C for 10 minutes; 94°C for 15 minutes; 10 cycles of 94°C for 20s, 65–57°C for 60s (dropping 0.8°C per cycle); 35–40 cycles 94°C for 20s, 57°C for 60s. Fluorescence detection was performed at room temperature using a BMG Pherastar® scanner fitted with Fl 485/520, Fl 520/560 and Fl 570/610 optic modules. Genotype calling was performed using the Kraken software package version 11.5 (LGC Genomics). Fluorescent intensity was normalised for pipetting volume using the ROX standard contained within the PACE-RT master mix.

### Data analysis

Data analysis was performed only on those samples for which 10 or more probes produced a genotype call. Samples were grouped into identical genotypes with the script qc_genotype_data.pl, which was added to the GITHUB (https://github.com/pr0kary0te/SARSmarkers) along with the SNP marker discovery pipeline.

## Results

### Minimal marker set

Up to week 18, the high-quality COG-UK sequence alignment comprised 14,277 sequences, as indicated in the accompanying metadata file. We found 41 SNPs meeting our criteria of a minimum minor allele frequency of 0.1%. Of these, our pipeline identified 22 as sufficient to provide the maximum possible discrimination between samples in the COG-UK dataset. Three SNPs were removed manually from this list as either their flanking sequences (for probe design) were overlapping or contained ambiguous bases (‘N’) close to the SNP of interest. Prior to wet-lab marker validation, we found that these 19 SNPs were capable of delineating 59 distinct variants from the COG-UK sequence alignment (S3 Table). To test the discriminatory power of the 19-marker set (hereafter, named the test set), random pairs of haplotypes for our marker positions were sampled from the COG-UK sequence alignment without replacement. We found that 89.1% of 6,202 random sample pairs were distinct at one of more marker positions. The flanking sequences for the 19 selected SNPs of the test set (S1 Table), were sent to 3CR Biosciences for probe design.

#### Synonymous and non-synonymous SNPs

All nineteen SNP markers in the test set target SNPs located in coding sequences. With regard to the codons within the open reading frame (ORF) of these genes, five of the SNPs were at position 1, six at position 2 and eight at position 3. Twelve of the SNPs were non-synonymous and would result in changes to the amino acid at the given position (Table 2).

**Table 2:** Alternative SNPs and their effect on protein coding.

Primer ID	Gene	Protein	Position	Alternative Codons		Syn. / Non-syn.	Alternative amino acids	
**Bris_SARS-CoV-2_313**	ORF1a	Nsp2	3	CTC	CTT	Syn	Leucine	----
**Bris_SARS-CoV-2_1059**	ORF1a	Nsp2	2	ACC	ATC	Syn	Threonine	----
**Bris_SARS-CoV-2_2416**	ORF1a	Nsp3	3	TAC	TAT	Syn	Threonine	----
**Bris_SARS-CoV-2_2558**	ORF1a	Nsp3	1	CCA	TCA	Non	Proline	Serine
**Bris_SARS-CoV-2_2891**	ORF1a	Nsp3	1	GCA	ACA	Non	Alanine	Threonine
**Bris_SARS-CoV-2_4002**	ORF1a	Nsp3	2	ACT	ATT	Non	Threonine	Isoleucine
**Bris_SARS-CoV-2_11083**	ORF1a	Nsp5	3	TTT	TTG	Non	Phenylalanine	Leucine
**Bris_SARS-CoV-2_14408**	ORF1ab	Nsp12	2	CTT	CCT	Non	Leucine	Proline
**Bris_SARS-CoV-2_14805**	ORF1ab	Nsp12	3	TAC	TAT	Syn	Tyrosine	----
**Bris_SARS-CoV-2_17247**	ORF1ab	Nsp13	3	CGT	CGC	Syn	Arginine	----
**Bris_SARS-CoV-2_19839**	ORF1ab	Nsp15	3	AAC	AAT	Syn	Asparagine	----
**Bris_SARS-CoV-2_20268**	ORF1ab	Nsp15	3	TTA	TTG	Syn	Leucine	----
**Bris_SARS-CoV-2_20578**	ORF1ab	Nsp15	1	GTG	TTG	Non	Valine	Leucine
**Bris_SARS-CoV-2_25350**	S	Spike	2	CCA	CTA	Non	Proline	Leucine
**Bris_SARS-CoV-2_25429**	ORF3a	Ap3a	1	GTA	TTA	Non	Valine	Leucine
**Bris_SARS-CoV-2_25563**	ORF3a	Ap3a	3	CAG	CAT	Non	Glutamine	Histidine
**Bris_SARS-CoV-2_27046**	M	Matrix	2	ACG	ATG	Non	Threonine	Methionine
**Bris_SARS-CoV-2_28144**	ORF8	Ap8	2	TTA	TCA	Non	Leucine	Serine
**Bris_SARS-CoV-2_28580**	N	Nucleoprotein	1	GAT	TAT	Non	Aspartate	Tyrosine

In the Alternative Codons columns, the codon with the predominant SNP in the COG-UK 2020-05-08 dataset is listed first. Position refers to the SNP position with respect to the in-frame codon. Abbreviations: Nsp = non-structural protein; Ap = accessory protein; Non = non-synonymous, Syn = synonymous.

#### Evaluation of the test set

Initial evaluation of the test set was performed using the two cell culture propagated SARS-CoV-2 isolates GBR/Liverpool_strain/2020 and hCoV-19/England/02/2020. The two virus genomes vary at ten nucleotide positions (Table 1) but have no differences in the wt spike gene sequences. However, in addition to the wt viral genome, the hCoV-19/England/02/2020 virus stock was known to contain a variant genome that arose during viral passage in tissue culture, which had a 24 nt in frame deletion in the spike gene sequence (BrisΔS, Table 1). Genotypes were obtained for all 19 markers (Table 3).

**Table 3:** Comparison of genotyping and sequencing data obtained for the test set.

Probe ID	wt Liverpool_strain		BetaCoV/England mix		Notes	COG-UK
	Genotype	Sequence	Genotype	Sequence		
Bris_SARS-CoV-2_313	C:C	C	C:C	C	Concord	C/T
Bris_SARS-CoV-2_1059	C:C	C	C:C	C	Concord	C/T
Bris_SARS-CoV-2_2416	C:C	C	C:C	C	Concord	C/T
Bris_SARS-CoV-2_2558	C:C	C	C:C	C	Concord	C/T
Bris_SARS-CoV-2_2891	G:G	G	G:G	G	Concord	G/A
Bris_SARS-CoV-2_4002	C:C	C	C:C	C	Concord	C/T
Bris_SARS-CoV-2_11083	T:T	T	G:G	G	Separation	G/T
Bris_SARS-CoV-2_14408	C:C	C	C:C	C	Concord	T/C
Bris_SARS-CoV-2_14805	C:C	C	C:C	C	Concord	C/T
Bris_SARS-CoV-2_17247	T:T	T	T:T	T	Concord	T/C
Bris_SARS-CoV-2_19839	T:T	T	T:T	T	Concord	T/C
Bris_SARS-CoV-2_20268	A:A	A	A:A	A	Concord	A/G
Bris_SARS-CoV-2_20578	G:G	G	G:G	G	Concord	G/T
Bris_SARS-CoV-2_25350	C:C	C	C:C	C	Concord	C/T
Bris_SARS-CoV-2_25429	G:G	G	G:G	G	Concord	G/T
Bris_SARS-CoV-2_25563	G:G	G	G:G	G	Concord	G/T
Bris_SARS-CoV-2_27046	C:C	C	C:C	C	Concord	C/T
Bris_SARS-CoV-2_28144	T:T	T	C:C	C	Separation	T/C
Bris_SARS-CoV-2_28580	G:G	G	G:G	G	Concord	G/T

Sequences “Concord” where the SARS-CoV-2 isolates GBR/Liverpool_strain/2020 and hCoV-19/England/02/2020 all share the same genotype and sequence. Separation denotes genotyping call differences between the two isolates confirmed by sequencing. Alleles in the last column are those reported in the COG-UK database (from the 2020-05-08 dataset COG consortium https://www.cogconsortium.uk/data/ (14,277 sequences) with the major/minor alleles.

#### Concordance between genotyping and sequencing

The two SARS-CoV-2 isolates GBR/Liverpool_strain/2020 and hCoV-19/England/02/2020 had been sequenced, enabling a comparison with our genotyping data (Table 3). All genotyping results were concordant with the sequence data. In two cases, it was possible to confirm SNPs (at nts 11083 and 28144) differentiating the two wt SARS-CoV-2 isolates with both sequence and genotyping data. We also compared these data with the available COG-UK sequences from the 2020-05-08 dataset (representing PCR positives samples circulating March–May 2020). This showed that the majority of genotype calls concord with the major allele found in the COG-UK database.

### Genotyping clinical SARS-CoV-2 samples

To further evaluate the test set we genotyped 50 SARS-CoV-2 positive samples obtained from PHE (samples collected from the South West of England). For 41 of the 50 samples, results were obtained from at least 50% of the SNP markers in our panel; those that fell below this threshold were excluded from further analysis (S4 Table). For 22 of the remaining 41 samples results were obtained for all 19 markers and for a further 13 samples, results were obtained from at least 15 of the 20 markers.

**Fig 3:**
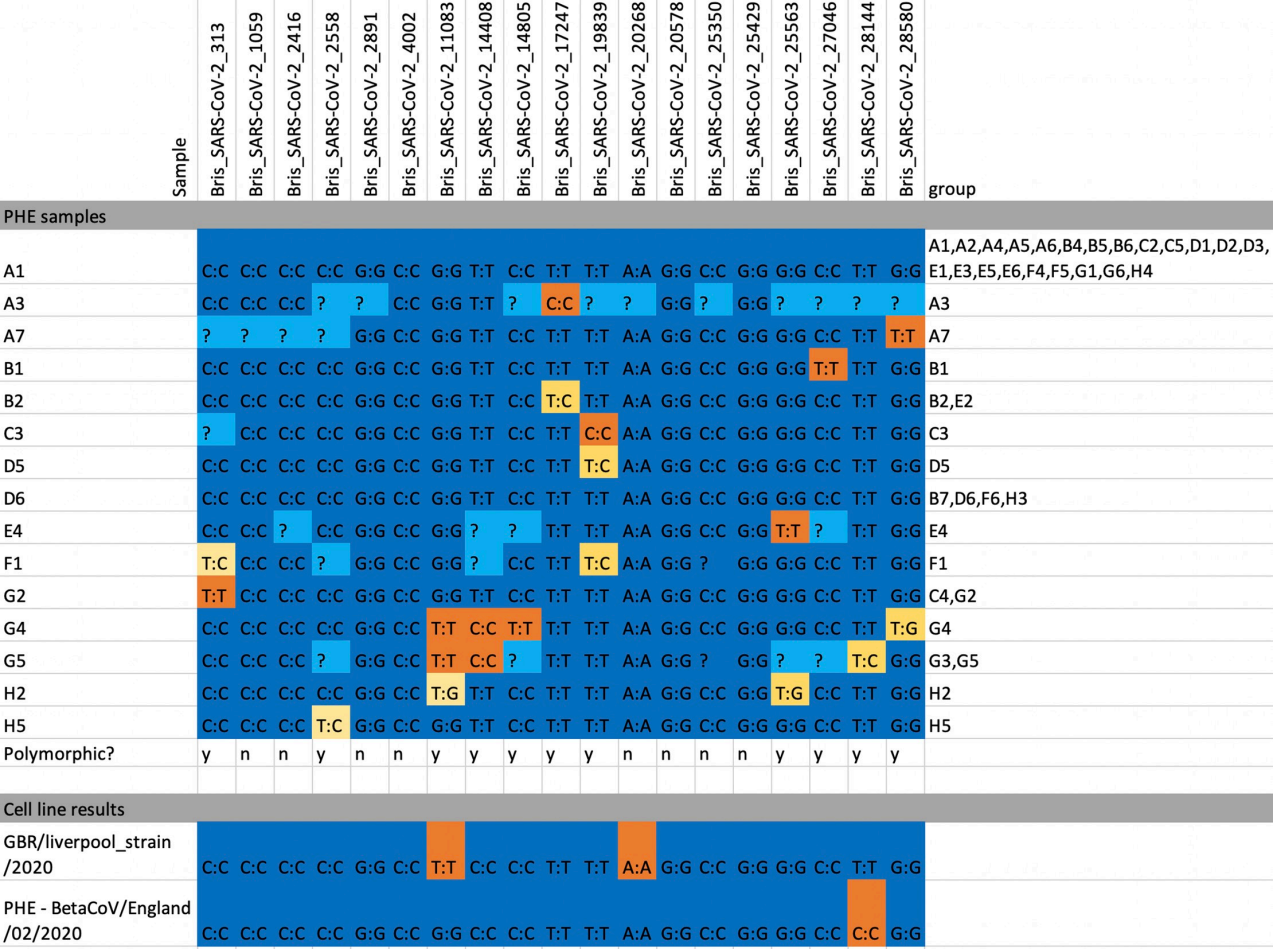
Genotyping calls for all samples. SNPs with a single allele call per sample are marked in dark blue (major allele) or orange (minor allele). Mixed calls are shown in gold and missing data in light blue. Twelve out of 19 markers were polymorphic in our small test panel of PHE samples and cell lines (eleven out of 19 markers were polymorphic in PHE samples) and eight samples had mixed calls for one or more markers.

We found that 11 of the 19 markers were polymorphic among the 50 PHE samples and could be used to assign them to 15 distinct groups (Fig 3 and S4 Table). To quantify the utility of our SNP panel in separating positive samples into distinct groups, we sampled random pairs of the 50 genotyped samples 1000 times and found that they were separated by at least one marker in 619 cases (61.9%).

#### Marker fail rate in PHE samples

The average fail rate by marker (that is, the marker produced no signal for some samples) was 19.4% ranging from 4% (marker Bris_SARS-CoV-2_25429) to 32% (markers Bris_SARS-CoV-2_2558 and Bris_SARS-CoV-2_25350). The number of fails per sample ranged from 0% (22 of the samples) to 80% (2 of the samples); those samples with fewer than 10 calls (9 in total) were removed from further analysis (S4 Table).

### An evolving target

The Microreact website [8] shows how SARS-CoV-2 lineage frequencies have changed during the outbreak and similarly the SNPs we targeted in our panel also changed in frequency over time. To quantify the effect of alterations in SNP frequency over time on the discriminative power of the 19 SNP panel, it was tested bioinformatically against random pairs of samples drawn from week 19 through week 35 in the 2020-09-03 COG-UK data. The probability of the original marker set discriminating a random pair of samples decreased from 89.1 to 77.6%. There was, however, an anomaly in this analysis as our G/T SNP at position 11,083, recorded as a variant in the 2020-05-08 COG-UK data and polymorphic in our genotyping results, is reported as the non-IUPAC character “?” the 2020-09-03 COG alignment due to it exhibiting homoplasy in phylogenetic reconstruction (Andrew Rambaut, personal communication). The loss of data for this marker from the latest COG-UK alignment means we will have underestimated the discriminatory power of our panel on more recent samples. Nonetheless, we re-ran the SNP marker discovery pipeline on the week 19–35 sequences and found that the number of SNPs present at a frequency greater than 0.001 had increased from 41 to 97 (noting that the SNP at 11,083 has been masked out of that alignment) and that 51 markers were now required to discriminate all samples to the maximum amount possible. However, the majority of variants were extremely rare, such that just the first 24 markers (S5 Table) were capable of discriminating 95% of randomly selected sample pairs.

## Discussion

Bioinformatic analysis of COG-UK sequence alignment data from May 2020 suggested that a small number of PACE-RT genotyping assays could provide useful viral genotype identification for UK SARS-CoV-2 positive samples. We developed a genotyping ‘test panel’ of 19 markers; initial evaluation of a set of two SARS-CoV-2 isolates (GBR/Liverpool_strain/2020 and hCoV-19/England/02/2020) showed that all of the markers designed produced distinct genotypes with low failure rates and comparison with available sequencing data confirmed the alleles identified in the test panel. These results were also the first demonstration of genotyping directly from an RNA virus in a single step assay.

We went on to test our panel on 50 qRT-PCR positive SARS-CoV-2 samples, that were collected across the UK in April 2020. Whilst a few of the PCR-positive samples we obtained from PHE did not produce results, for the majority of our test marker panel all of the markers themselves performed as expected, with missing data being attributable to low quality nasopharyngeal swabs samples rather than with any particular markers. Eight of the nineteen markers were not polymorphic in the clinical samples we were able to obtain, which was not unexpected given the small sample size. Whilst we have no reason to assume that these eight markers are not capable of producing polymorphic calls, we were unable to obtain any further samples to test this during our study. The 50 samples could be split into 15 distinct genotypes based on the genotyping data obtained and there was a 61.9% probability that any two randomly chosen samples from our set of 50 would have a distinct genotype. This is lower than the predicted discriminatory power of the panel (89.1%) and can be explained by missing data for some sample/marker combinations, resulting from us having access to very limited quantities of PCR-positive samples, which at the time proved to be in high demand locally for validation of qPCR assays. In a standard diagnostic laboratory workflow, more RNA would be available following qPCR positive sample results and could be used in a genotyping screen to identify variants.

Genotyping can detect mixed viral samples. We found that eight of the 50 PHE samples had mixed calls, with B2, E2, D5, G4, G5, H5 mixed at one SNP and F1 and H2 both mixed for two. We interpret this as possible evidence of infection by two genotypes, differing in at least one or two SNPs respectively. The ability of PACE-RT genotyping to correctly cluster genotypes improves as larger number of samples are processed, so it is possible that some mixed calls would resolve to single-allele calls as more samples were added to this pilot-scale dataset.

A limitation of genotyping is the ascertainment bias of the probe design. Novel mutations cannot be detected which relies on an existing sequencing effort such as that performed by the COG-UK Consortium. As new mutations are discovered by traditional sequencing, the tools made available in our software pipeline may be used to design a relevant probe set for the current circulating viral population. This requires the removal of redundant probes and introduction of some novel probes; involving primer design and optimisation using samples previously sequenced to obtain clear amplification of probes in a positive control and the absence of amplification in a non-target negative control.

Markers in the panel were updated based on variant analysis of the 2020-09-03 release of sequences from the COG-UK consortium to reflect the new variants circulating in the UK. We found 91 SNPs with a frequency > 0.01 in the week 19–35 analysis, compared to 41 SNPs in the data to week 18. The majority of the SNPs were rare, however, and we found that limiting the marker set to the most informative 24 markers gave us slightly better discriminatory power on the week 19–35 samples (95% of random pairs differentiated) than our original 19 marker set designed from week 1–18 data (89% differentiated). SNPs will continue to arise and go extinct, but our analysis suggests that a small and cost-effective panel of 20–24 markers will continue to provide useful discriminatory power in many settings. Our SNP panel was designed using sequence data from across the UK. It would be equally possible to use the global data from www.gisaid.org to design a set of markers for global surveillance, or to focus on a specific region of the UK for panel design if desired: input sequences would simply be pre-filtered using the sample region from the COG metadata file. Like other screening and genomic surveillance tools it is necessary to update and optimise the SARS-CoV-2 genotyping panel as the virus evolves. Bespoke variant specific genotyping panels could also be designed to track specific variants of concern, for example the B1.1.7 or B1.351 variants and could provide a rapid approach to monitor variants.

While sequence data may offer a greater depth of information, PACE-RT genotyping can offer a rapid and low-cost solution to rapidly identify sample differences within a population. A set of 20–24 markers may be screened against 192 samples for around £2.30 per sample and savings are possible as sample numbers increase beyond this.

Genotyping is highly scalable and suited to a high throughput setting but does not require bespoke equipment which makes it suitable as an additional screening method in diagnostic testing facilities. The methods described here may be performed with only a thermocycler and FRET-capable plate reader such as that found within RT-PCR instruments. To scale up, a small laboratory equipped with a 1536-well plate thermocycler and fluorescent plate-reader along with sample handling robotics and sample tracking LIMS such as KRAKEN should be able to genotype several thousand positive samples per day with input from a single trained operator. As the data may be exported simply in an excel spreadsheet format, complex data analysis pipelines are not required to obtain the genotype scores.

## Conclusion

To date, SARS-CoV-2 variants have been determined by sequencing positive samples with only a small proportion of PCR samples assessed; as of 9^th^ October 2020 there were 36,593,879 reported global cases of COVID-19 and 141,000 viral genomic sequences deposited on GISAID [15]. Our results show that PACE-RT genotyping with a small panel of SNPs can add useful genotype information to PCR-positive samples at a low cost. The fast turnaround of this approach coupled with the ease with which it can be automated means that it has the potential to provide additional detail for epidemiological studies. It is not, however a substitute for continued sequencing. Rather, the two approaches are complementary and genotyping panels will need to be cross checked against sequence alignments at regular intervals to ensure that new mutations are included and that loci which have become fixed or nearly so, are replaced. At the time of writing, it is not possible to sequence every PCR positive sample in the UK and genotyping has the potential to add genotype information to all positive results with minimal investment in equipment for diagnostic testing laboratories at very low cost per sample. Testing laboratories may also consider designing their own marker panels based on regional or national datasets (the latter in our case) to maximise the fit between sample SNP frequencies and the panel. Our primer design pipeline is freely available for this purpose. The advantage of PACE-RT technology is that the SNP panel can be modified at low cost on a regular basis; in a medium to high-throughput laboratory the cost of new primer sets would not be a significant factor. The only real limitation of our approach is that it is not necessarily possible to assign samples to a specific named lineage in the way that full sequence data allows. We have shown, however that there is a high probability (61.9%) of being able to separate any two samples into distinct genotypes using our marker panel, and in many settings this will be sufficient to identify or rule out transmission routes and thus inform public health policy to minimise the spread of the virus. With the recent emergence of several variants of concern and potential importance in the context of vaccine deployment, this approach could provide a useful tool for epidemiological surveillance.

## Supporting information

S1 Table Test panel SNPs with flanking sequences. (CSV)

S2 Table Primer sequences for test panel. (XLSX)

S3 Table Regional haplotypes. (CSV)

S4 Table PHE 30-09-2020 genotypes. (XLSX)

S5 Table Markers weeks 19–35. (CSV)

S1 File COG-UK authorship. (DOCX)

## References

[1] Organisation WH. Timeline: WHO’s COVID-19 response 2020 [cited 2020 7/10]. Available from: https://www.who.int/emergencies/diseases/novel-coronavirus-2019/interactive-timeline/].

[2] An integrated national scale SARS-CoV-2 genomic surveillance network. Lancet Microbe. 2020;1(3):e99–e100. 10.1016/S2666-5247(20)30054-9 32835336 PMC7266609

[3] Elbe S, Buckland-Merrett G. Data, disease and diplomacy: GISAID’s innovative contribution to global health. Glob Chall. 2017;1(1):33–46. 10.1002/gch2.1018 31565258 PMC6607375

[4] Taiaroa G, Rawlinson D, Featherstone L, Pitt M, Caly L, Druce J, et al Direct RNA sequencing and early evolution of SARS-CoV-2. bioRxiv. 2020:2020.03.05.976167.

[5] Callaway E. The coronavirus is mutating—does it matter? Nature. 2020;585(7824):174–7. 10.1038/d41586-020-02544-6 32901123

[6] Boivin S, Cusack S, Ruigrok RW, Hart DJ. Influenza A virus polymerase: structural insights into replication and host adaptation mechanisms. J Biol Chem. 2010;285(37):28411–7. 10.1074/jbc.R110.117531 20538599 PMC2937865

[7] Kupferschmidt K. Mutations can reveal how the coronavirus moves—but they’re easy to overinterpret. Science. 2020 3 9, 2020.

[8] Argimón S, Abudahab K, Goater RJE, Fedosejev A, Bhai J, Glasner C, et al Microreact: visualizing and sharing data for genomic epidemiology and phylogeography. Microbial Genomics. 2016;2(11). 10.1099/mgen.0.000093 28348833 PMC5320705

[9] Hadfield J, Megill C, Bell SM, Huddleston J, Potter B, Callender C, et al Nextstrain: real-time tracking of pathogen evolution. Bioinformatics. 2018;34(23):4121–3. 10.1093/bioinformatics/bty407 29790939 PMC6247931

[10] Rambaut A, Holmes EC, O’Toole Á, Hill V, McCrone JT, Ruis C, et al A dynamic nomenclature proposal for SARS-CoV-2 lineages to assist genomic epidemiology. Nature Microbiology. 2020;5(11):1403–7. 10.1038/s41564-020-0770-5 32669681 PMC7610519

[11] Stevens EL, Timme R, Brown EW, Allard MW, Strain E, Bunning K, et al The Public Health Impact of a Publically Available, Environmental Database of Microbial Genomes. Front Microbiol. 2017;8:808 10.3389/fmicb.2017.00808 28536563 PMC5422427

[12] Davidson AD, Williamson MK, Lewis S, Shoemark D, Carroll MW, Heesom K, et al Characterisation of the transcriptome and proteome of SARS-CoV-2 using direct RNA sequencing and tandem mass spectrometry reveals evidence for a cell passage induced in-frame deletion in the spike glycoprotein that removes the furin-like cleavage site. bioRxiv. 2020:2020.03.22.002204. 10.1186/s13073-020-00763-0 PMC7386171 32723359

[13] Boom R, Sol CJ, Salimans MM, Jansen CL, Wertheim-van Dillen PM, van der Noordaa J. Rapid and simple method for purification of nucleic acids. J Clin Microbiol. 1990;28(3):495–503. 10.1128/JCM.28.3.495-503.1990 1691208 PMC269651

[14] 3CRBioscience. Assay Design 2020 [Available from: https://3crbio.com/free-assay-design/].

[15] Dong E, Du H, Gardner L. An interactive web-based dashboard to track COVID-19 in real time. Lancet Infect Dis. 2020;20(5):533–4. 10.1016/S1473-3099(20)30120-1 32087114 PMC7159018

